# Children and adolescents adjustment to parental multiple sclerosis: a systematic review

**DOI:** 10.1186/1471-2377-14-107

**Published:** 2014-05-19

**Authors:** Neda Razaz, Reza Nourian, Ruth Ann Marrie, W Thomas Boyce, Helen Tremlett

**Affiliations:** 1 School of Population and Public Health, Faculty of Medicine, University of British Columbia, 2206 East Mall, Vancouver, British Columbia, V6T 1Z3, Canada; 2 Departments of Internal Medicine and Community Health Sciences, University of Manitoba, Health Sciences Centre, GF 543-820 Sherbrook Street, Winnipeg, Manitoba, R3A 1R9, Canada; 3 Division of Developmental-Behavioral Pediatrics, Department of Pediatrics, University of California, 3333 California Street, Suite 245, San Francisco, CA 94118, USA; 4 Brain Research Centre and Department of Medicine (Division of Neurology), Faculty of Medicine, University of British Columbia, Vancouver, Canada; 5 Vancouver Coastal Health Research Institute, S178 Koerner Pavilion, 2211 Wesbrook Mall, Vancouver, British Columbia, V6T 2B5, Canada

**Keywords:** Multiple sclerosis, Child development, Parenting, Child of impaired parents, Cohort studies

## Abstract

**Background:**

Families are the primary source of support and care for most children. In Western societies, 4 to 12% of children live in households where a parent has a chronic illness. Exposure to early-life stressors, including parenting stress, parental depression and parental chronic disease could lead to harmful changes in children’s social, emotional or behavioural functioning. Little is known about the child living with a parent who has Multiple Sclerosis (MS). We systematically reviewed the literature regarding possible effects of having a parent with MS on the child’s or adolescent's psychosocial adjustment.

**Methods:**

The following databases: MEDLINE, PsychInfo, CINAHL, EMBASE, Web of Knowledge, ERIC, and ProQuest Digital Dissertations were searched (from 1806 to December 2012). References from relevant articles were also manually searched. Selected studies were evaluated using the Graphic Appraisal Tool for Epidemiology (GATE).

**Results:**

The search yielded 3133 titles; 70 articles were selected for full text review. Eighteen studies met inclusion criteria. Fourteen studies employed quantitative techniques, of which 13 were cross-sectional and one was longitudinal. Four studies were both qualitative and cross-sectional in design. Only 2 of 18 studies were rated as having high methodological quality. Overall, eight studies reported that children of MS patients exhibited negative psychosocial traits compared with children of “healthy” parents. Specifically for adolescents, greater family responsibilities were linked to lower social relationships and higher distress. Three studies indicated that parental MS was associated with positive adjustment in children and adolescents, such as higher personal competence, while four found no statistically significant differences.

**Conclusion:**

Although having a parent with MS was often reported to have negative psychosocial effects on children and adolescents, there was a lack of consensus and some positive aspects were also found. However, few high quality studies were identified which makes it difficult to draw evidence-based conclusions at this point. There are potentially important, long-term impacts of early life stressors, such as having a parent with a chronic disease, on subsequent life chances and health, and thus more extensive and higher quality research in this area is greatly needed.

## Background

In Western societies, 4 to 12% of children and adolescents aged 18 and under live in households where a parent has a chronic illness [1-3]. Increasing evidence suggests that having a parent with a chronic condition can put children at a higher risk of developing emotional and behavioural difficulties due to changes in parent–child interactions [3].

Multiple sclerosis (MS) is a chronic degenerative disease of the central nervous system and is the most common non-traumatic cause of neurological disability among young adults in the Western world [4]. MS typically manifests between the ages of 20 and 40 years, at a life stage when parenting is an important issue for many [5]. Interestingly, reproductive decision-making by people with MS seems to follow the same pattern as the general population [6]. Consequently, many children are exposed to a parent trying to cope with a potentially disabling chronic condition. MS is a particularly challenging disease, and the unpredictable and variable clinical course can cause considerable stress and anxiety on patients and their families [7,8].

While much research and resources now focus on the child or adolescent who has MS, less is known about the child living with a parent who has MS [9,10]. We aimed to systematically review the literature to address the question - *what are the possible effects on children and adolescents*’ *psychosocial adjustment of having a parent with MS*? In doing so, we aimed to illuminate the impact of parental health issues on children’s development, and open avenues for early identification and potential preventive interventions.

## Methods

### Search strategy

A comprehensive search of the literature was undertaken in December 2012, accessing the following databases: MEDLINE, PsychInfo (from 1806), CINAHL (from 1982), EMBASE (from 1974), Web of Knowledge (from 1900), ERIC (from 1966), and ProQuest Digital Dissertations (from 1980). Search terms included ‘Multiple Sclerosis’ , ‘family’ , ‘Parents’ , ‘Parent–child Relations’ , ‘Child of Impaired Parents’ , ‘Nuclear Family’ and ‘Caregivers’ (see Additional file 1 online for detailed strategy). References from identified articles were also hand searched for potentially relevant articles. Although we searched selected conference proceedings for emerging research, specifically the 2010, 2011 and 2012 proceedings from the annual meetings of the American Academy of Neurology and the European and American Committees of Treatment and Research in Multiple Sclerosis (the largest conferences covering MS research), we did not find any relevant abstracts to include in our data synthesis.

### Inclusion and exclusion criteria

Only original full-text peer-reviewed published studies fulfilling the following criteria were included (1) school-aged children or adolescents, under the age of 18 years, were part of the study sample; (2) at least one parent was diagnosed with MS; (3) evaluated potential factors associated with parental MS and psychosocial adjustment in children and adolescents was included, regardless of the actual direction of findings (positive, negative or neutral); (4) findings were reported using statistical or qualitative analysis; and (5) were published in English. Non-empirical studies were excluded (i.e. clinical reports, reviews, comments, experiences, case studies, or opinions).

### Data collection process

Two individuals independently screened the titles and abstracts of all identified studies (N.R. & R.N.). All studies considered eligible underwent a full-text review by one reviewer (N.R.). Data extraction was conducted using a pre-piloted form, which captured: study design, sample size, duration of exposure (to MS), outcomes measured, main findings and methodological quality (see Table 1). Accuracy of data abstraction was cross-checked and confirmed, on a random sample of 10 studies out of the 70 studies that underwent full-text review, by a second reviewer (R.N.). The level of agreement between the two reviewers was 90% and any disagreements were resolved through consensus.

**Table 1:** Summary of studies examining exposure to parental MS and psychosocial adjustment in children and adolescents

**Author/Year**	**Country**	**Study design**	**Sample (age range of children)**	**A. Exposure to parental MS B. Parental MS duration**	**Outcomes measured**	**Evaluator**	**Main findings**	^*^Quality[11]
Arnaud 1959 [12]	United States	Quantitative/Cross-sectional	60 children with an MS parent and 221 with a “healthy” parent(s) (7–16 years)	A. Mean = 7.2 years (SD: 2.5) B. Range: 3–17 years	^a^Psychological characteristics:	Third Party: Author	Children with a parent with MS scored higher in: Body concerns Dysphoric feelings, Hostility, Constraint in interpersonal relations, Dependency needs	Medium
					(1) General anxiety			
					(2) Body concern			
					(3) Dysphoria			
					(4) Hostility			
					(5) Constraint in interpersonal relations			
					(6) Dependency longings			
					(7) False maturity			
Blackford 1999 [13]	Canada	Qualitative/Cross-sectional	22 children with an MS parent. No comparison group.	Did not specify	Children’s descriptions of life with a parent who has MS	Third Party: Author	Children with an MS parent described higher personal competence, hopefulness, and spirituality. Negative factors that children encountered were attributable more to society than to their parent’s condition.	Low
Bogosian 2011 [14]	UK	Qualitative/Cross-sectional	15 children with an MS parent (13-18 years). No comparison group.	Did not specify	Interviews were conduced asking	Third Party: Trained Interviewer	Adolescents described both positive and negative experiences related to having a parent with MS. Benefits to having a parent with MS included reports of feeling more empathetic to others and more grown-up. Negative impacts included family tension, less time to spend with friends, and worries about the future.	High
					● What is it like for you to have a parent with MS			
					● How does your mum’s/dad’s MS affect your?			
					a. Social life			
					b. Family life			
Brandt 1998 [15]	Unites States	Quantitative/Cross-sectional	174 children with an MS parent (7-17 years). Population ‘norms’ as comparison group.	Did not specify	^b^Children’s Mental Health	Parent without MS	25% of children in this study (45 of the 174) were classified as being “at risk” for a mental health problem compared with the rate of the prevalence rate in the general child population of 12% to 20%.	Low
Crist 1993 [16]	United States	Quantitative/Cross-sectional	31 girls with mothers with MS and 34 girls with “healthy” mother(s) (8-12 years)	A. Minimum = 2 years	Mother-daughter interactions during a work task and a play task assessed as: receptiveness, directiveness, and dissuasiveness	Third party: Author	Similar proportions of receptive, directive, and dissuasive behaviors were used by mothers with MS and their daughters compared with those used by control group mothers and their daughters.	Medium
				B. Range: 2 - 28 years				
De Judicibus 2004 [5]	Australia	Quantitative/Cross-sectional	48 children with an MS parent (4–16 years). No comparison group.	B. Mean = 5.6 years (ranged: 1- 19 years)	^c^Children’s emotional and behavioural well-being	Parent with MS	Children with an MS parent demonstrated more difficulties in how they related to others, the distress they experienced and how they managed their lives. However, they did not reveal higher levels of clinical symptoms requiring treatment.	Low
Diareme 2006 [7]	Greece	Quantitative/ Cross-sectional	56 children with an MS parent and 64 with a “healthy” parent(s) (4–17 years)	B. Mean = 10.3 years (SD: 9.5)	^d, e^Children’s emotional and behavioural problems	Child	Children whose parents, especially mothers, had MS presented greater emotional and behavioural problems than comparison children. Children’s problems were positively associated with maternal depression and family dysfunction. Family dysfunction predicted children’s overall and externalizing problems, while the severity of impairment of the MS mother predicted children’s internalizing problems.	Medium
Kikuchi 1987 [17]	Canada	Qualitative/Cross-sectional	32 children with an MS parent (6 - 17 years). No comparison group.	Did not specify (although at the time of MS diagnosis subjects ranged from newborns to 15 years; mean = 6.5 years)	Children reported quality of life	Third Party: Trained Interviewer	For most part children reported a good quality of life. Although, children expressed limited knowledge of MS and feelings of fear, anger and sadness.	Medium
Olga 1974 [18]	United States	Quantitative/ Cross-sectional	124 children with an MS parent and 60 with a “healthy” parent(s) (7–11 years)	A. Minimum = 2 years	^f^Body image	Child	Body image scores did not differ between groups	Low
							Body image distortion tended to be greater in girls with MS mothers than girls with MS fathers or boys with MS mother	
Pakenham 2006 [19]	Australia	Quantitative/ Cross-sectional	48 children with an MS parent and 145 with a “healthy” parent(s) (10–25 years)	B. Mean = 9 years (SD: 7; range: 4 months to 29 years)	Children’s positive (benefit finding, life satisfaction and positive affect) and negative (distress and health status) adjustment	Child	Children with a parent with MS had poorer adjustment, greater family caregiving responsibilities and lower levels of life satisfaction and positive affect	Low
Pakenham 2012 [20]	Australia	Quantitative/ Longitudinal	Time 1: 130 children with an MS parent (10-20 years) Time 2 (After 12 months): 91 children with an MS parent (10-20 years). No comparison group.	At time 1:B. Mean = 8.2 years (SD: 5.8; range: 4 months to 25 years)	^g^Children’s negative (behavioural emotional difficulties, somatisation) and positive (life satisfaction, positive affect, prosocial behaviour) adjustment	- Child - Parent with MS - Parent without MS	At time 1 higher total caregiving was associated with lower life satisfaction and higher somatization and total difficulties. Higher total difficulties were also associated with greater social-emotional care. At time 2, higher caregiving responsibility was associated with lower life satisfaction and higher total caregiving was associated with increased prosocial behaviour. Further, time 1 instrumental and social-emotional care domains were associated with poorer time 2 adjustment.	Low
Paliokosta 2009 [21]	Greece	Quantitative/Cross-sectional	56 children with an MS parent (4-17 years). No comparison group.	B. Mean = 10.3 years (range = 2 months to 21 years)	^b^Children’s mental health and behaviour	Third Party: Trained Interviewer - Parent with MS - Parent without MS - Child	Children and adolescents who had “partial information” about parental MS presented with higher scores in social difficulties and internalizing behaviours as well as higher total problems on the child behaviour checklist. They also presented with higher score on social problems.	Low
					● Interviews were also conducted with the child and the parent about the amount of information regarding parental MS given to child			
Peters 1985 [22]	Canada	Quantitative/Cross-sectional	33 children with a MS parent and 33 with a “healthy” parent(s) (12–18 years)	B. Mean = 9.2 years (range: 1.6 - 17.7 years)	^h^Family cohesion, expressiveness, conflict, independence, achievement orientation, intellectual-cultural orientation, active-recreational, moral-religious emphasis, organizations and control in a family	Child	Children of MS parents showed significant differences in the perception of their family environment v.s children of ‘healthy’ parents. Lack of ‘feeling of togetherness’ was reported	Medium
Steck 2005 [23]	Switzerland	Quantitative/Cross-sectional	41 children with an MS parent (6 – 18 years). No comparison group.	A. Mean = 3.5 years (for children < 12); mean = 8.2 years (for children > 12)	Children’s indication for psychotherapy	Third party: Trained Interviewer	Half of the children were estimated to benefit from individual psychotherapy aimed at enhancing ability to cope with the parental MS.	Low
Steck 2007 [8]	Germany, Greece, Switzerland	Quantitative/Cross-sectional	192 children with an MS parent (Mean = 9.8 years; SD: 4.8). No comparison group.	B. Mean = 6.5 years for MS fathers; Mean = 7.7 years for MS mothers	^b^Children’s mental health and behaviour	- Parent with MS Parent without MS Child	MS parents, especially mothers, as well as depressed mothers, or depressed “healthy” parents evaluated their children’s mental health problems with a higher prevalence within the internalizing spectrum. If two parents presented a depressive state, the prevalence of relevant psychological internalizing symptoms was twice or three times as high as the age norms.	Low
Turpin 2008 [10]	Australia	Qualitative/Cross-sectional	8 children with an MS parent (7–14 years). No comparison group.	Did not specify	Children’s day-to-day lives, their perceptions of their parent’s condition and their thoughts about the future	Third Party: Occupational therapist and a psychologist	Children described taking on additional roles and responsibilities that restricted their participation in developmentally appropriate occupations. Additional responsibilities can enhance children’s skills and provide pride and stress.	High
Yahav 2005 [24]	Israel	Quantitative/Cross-sectional	56 children with an MS parent and 156 with a “healthy” parent(s) (10–18 years)	A. >6 months	● A sense of personal concern and responsibility towards parents	Third Party: Trained Interviewer	Children of parents with MS felt more responsibility and obligation than children of healthy parents. They also exhibited higher degree of responsibility, more fear and anxiety related to MS, a greater sense of burden and a greater degree of anger.	Medium
					● Degree of responsibility and active protection of parents			
					● Fear and anxiety about parents’ future			
					● Burden of tasks and errands at home			
					● Anger			
Yahav 2007 [25]	Israel	Quantitative/ Cross-sectional	56 children with an MS parent and 156 with a “healthy” parent(s) (10–18 years)	A. >6 months	^e^Children’s emotional health and problem areas: delinquent behavior, aggression, attention problems, thought disorders, social acceptance problems, anxiety and depression, somatic complaints, and withdrawal behavior.	Third Party: Trained Interviewer	Children with an MS parent displayed higher levels of depression and anxiety than children from the control group. Furthermore, children in the study group reported a greater degree of separation anxiety, compared with the control group.	Medium

*Graphic Appraisal Tool for Epidemiology (GATE).

Instruments used to measure the stated outcomes:

^a^Rorschach test [26].

^b^Child Behaviour Checklist [27].

^c^Strengths and Difficulty Questionnaire [28].

^d^Achenbach’s Child Behaviour Checklist and Youth Self Report [29].

^e^Youth Self Report, [30] and Separation Individuation Test of Adolescence [31].

^e^Draw-A-Person [32], Semantic Differential [33] and The Body-Cathexis Scale [34].

^g^Youth Activities of Caregiving Scale [35].

^h^Family environment scale [36].

### Quality appraisal for included studies

No standard exists for conducting quality appraisals for observational studies in the context of a systematic review, so we adapted the Graphic Appraisal Tool for Epidemiology (GATE) [11], and supplemented this tool with topic-specific criteria (see Additional file 2 online), to assess both qualitative and quantitative studies. Each study received a summary quality score of low, medium, or high. It should be noted that a low quality score does not negate the contribution of a given study, especially in an emerging field where methods may not be well developed, but reflects methodological rigor in the context of all observational studies.

Due to heterogeneity in outcomes and methodologies in the selected studies, a meta-analysis was not possible; therefore a narrative analysis of data was conducted, with studies broadly grouped into those finding a negative, positive or no measureable effect on the developing child living with a parent who has MS, as well as by study design (e.g. quantitative vs qualitative and use of a comparison group).

## Results

### Literature search

**Figure 1:**
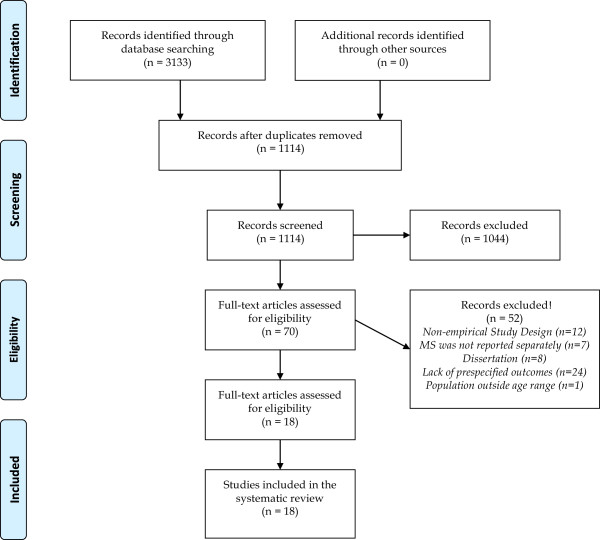
Search results and publication selection procedures.

The initial search returned 3133 citations, with 1114 remaining after duplicates were removed. Of these, 1044 articles were excluded at the title/abstract screening level for not fulfilling study criteria. Seventy articles underwent full-text review of which 52 were excluded for the following reasons: 1 included a population entirely outside the specified age range, 12 had a non-empirical study design, 7 included parents with a range of chronic conditions, without separating out MS, 8 were dissertation abstracts and 24 articles did not focus on psychosocial outcomes. Eighteen studies met the inclusion criteria (Figure 1) and were published between 1959 and 2012.

### Description of included studies

Of the 18 eligible studies (see Table 1), locations of study participants were as follows: United States (n = 4) [12,15,16,18], Canada (n = 3) [13,17,22], Switzerland (n = 2, 1 of which also included Germany and Greece) [8,23], Greece (n = 2) [7,21], UK (n = 1) [14], Israel (n = 2) [24,25], Australia (n = 4) [5,10,19,20]. Cohort sizes ranged from 8 [10] to 281 [12], with a total of 2051 children or adolescents studied overall. There were a higher number of mothers with MS than fathers, which could be partly attributable to the higher prevalence of MS in females than males or to mothers being more interested in participating in studies related to children. Most participants were of Caucasian/European-American descent.

### Measurement of the exposure

None of the 18 studies evaluated in this review specified the diagnostic criteria used for ascertaining MS cases. All study participants were recruited mainly through university neurology departments or specific national or local MS society centres. MS-specific clinical information on the affected parent were noted in some studies, with 2 providing a quantitative measure of disability (based on Kurtzke’s Expanded Disability Status Scale [EDSS] [37] score), with the affected parents ranging from an EDSS of 2 to 7, indicating ‘slight weakness’ through to ‘restricted to a wheelchair’ [16,23]. A further 9 studies provided other means of quantifying impairment, again with parents ranging from minimal impairment to wheelchair bound [7,8,10,14,15,17,19-21]. In the two studies reporting the affected parents’ disease course, most had relapsing-remitting MS [5,14]. Only six studies had enforced a minimum time of exposure to parental MS before enrollment [7,16,18,21,24,25], the rest did not specify or only mentioned parents’ disease duration.

### Quality and methodological challenges of included studies

Of the 18 studies included, 2 were considered of high quality, 7 moderate and 9 of low quality. Fourteen studies employed quantitative techniques, of which 13 were cross-sectional and one was longitudinal (and prospective) [5,7,8,12,15,16,18-25]. Four studies were both qualitative and cross-sectional in design [10,13,14,17]. Eight studies had a comparison group of children with ‘healthy’ parents [7,12,16,18,19,22,24,25]; the remainder had no control group.

### Outcome measures

A broad range of outcomes were considered in the selected studies, including, anxiety, depression, peer relations, caregiving responsibility, family cohesion, body image, parent–child interaction and hopefulness. Some studies used validated, standardized questionnaires such as Child Behavioural Checklist or Youth Self Report [29] and others used study-specific questionnaires to measure the outcome. Outcomes were assessed either by an interviewer who administered the questionnaires or were self-reported by the parents, the children or both. Amongst studies that systematically evaluated this question, 8 found a negative association between exposure to parental MS and adjustment in their offspring. Five studies did not find an association and 5 studies found both positive and negative effects of caring for a parent with MS.

### Negative psychosocial aspects

#### Quantitative studies with a comparison group

Of the 8 studies with a suitable comparison group, 6 described negative psychosocial outcomes for children who had a parent with MS compared with the children of “healthy parents”. Of these, two found higher levels of depression and anxiety [24] and greater emotional and behavioural problems [7] in the children with an MS-affected parent. Both studies were of medium quality. Studies measuring the caregiving activities of children with an MS parent highlighted that these children had more responsibility and obligations compared with those children with healthy parents and consequently a greater sense of burden, anger and lower levels of life satisfaction [19,25]. One study was scored low quality, the other medium. Furthermore, adolescents with an MS parent exhibited a higher degree of responsibility and experienced more fear and anxiety compared with adolescents with healthy parents [24]. Higher conflict, lower cohesion and a general ‘lack of togetherness’ was reported by children with an MS parent compare with children of non-MS parents in one study [22] and higher levels of body concern, hostility constraint, interpersonal relations and a pattern of false maturity in another [12]. Both were of medium quality.

Several studies compared the children of MS parent’s psychosocial score to the general population norm [8,15]. One study estimated that 25% (45 of 174) of children with MS parents were classified as being “at risk” for a mental health problem compared with 12 to 20% of children in the general population [15]. Furthermore, adolescent’s self-reported scoring for internalizing disorders was significantly over the expected normal [8]. Both were rated as low quality studies due to the failure to adjust for important demographic variables, such as SES and having suboptimal data collection.

### Quantitative studies with no comparison group

Of the remaining three quantitative studies with no comparison group, one demonstrated that children of parents with MS were at risk of mental health problems and would benefit from individual psychotherapy [23]. One study suggested that children who had partial information about their parent’s condition exhibited significantly more problems as compared with children who had explicit information or no information [21]. In addition one longitudinal study found that youths with greater caregiving responsibilities reported lower life satisfaction, higher somatization and higher emotional and behavioural difficulties [20]. All three studies were of low methodological quality, with interpretation of findings sometimes difficult in the light of no comparator group.

### Qualitative studies

Three of the four qualitative studies included described both positive and negative experiences related to having a parent with MS [10,14,17]. Higher family tension and extra responsibilities which limited children’s involvement with peers and time spent at play and learning were associated with having a parent with MS [10]. Further, all children expressed anxiety about both the immediate and long-term health and well-being of their parents [10,14]. Both studies were high quality. Last, a medium quality study noted that children’s limited knowledge and understanding of the disorder and the related implications of having a parent with MS seemed to be a threat to their achievement of happiness [17].

### Neutral (or no measurable) psychosocial effects

In contrast to some of the findings above, no statistically significant differences on body image distortion were found in children with an MS parent vs. a “healthy parent” [18]. Furthermore, no significant differences on mother-daughter interactions during work and play tasks were observed when the mother had MS vs. “healthy” mothers and daughters [16]. One study was of low quality [16], the other medium [18]. In addition, two studies showed that these children did not appear to differ from the community norms for overall difficulties and externalizing problems [5,8]. Yet these children were over three times more likely than a community sample to be perceived by their affected parents as having psychological problems. This might not be due to the child’s actual psychological well being, rather could relate to the parents’ perception of their own MS [5,8]. Both studies were of low quality. Last, a qualitative study (of medium quality) noted that for the most part, children with an MS parent reported a good quality of life [17].

### Positive psychosocial aspects

In one quantitative study, findings indicated that although parental MS was associated with a higher social-emotional burden, and a greater share of domestic-household duties, this actually lead to an increased in pro-social behaviour in youth [20]. Furthermore, these youth voiced a source of pride when taking on family responsibilities [10,17]. Children described having higher personal competence and feeling more empathetic to others and more “grown-up”, as the benefits to having a parent with MS [13,14].

## Discussion

In this systematic review we evaluated the association between parental MS and adjustment in children and adolescents. Overall, while most studies tended to report that children of MS patients exhibited negative psychosocial behaviour compared with children of “healthy” parents, some positive aspects in caring for a parents with MS were also highlighted. However, overall the strength of the evidence was rather weak, with only 2 of 18 studies rated as ‘high quality’ , which makes it difficult to draw evidence-based conclusions [10,14].

Nonetheless, our findings are broadly consistent with other systematic reviews, which also report negative psychosocial effects in children living with a parent with a physical disability or a chronic illness [3,38,39]. A meta-analysis looking at children who have a parent with a chronic illness found that overall these children displayed significantly more internalizing related behaviour (i.e. anxiety, depression, withdrawal) than children with healthy parents [3]. Furthermore, in a population-based sample of children with a parent dealing with a serious physical illness, there was an elevated risk of psychosocial maladjustment, with internalizing problems being more prevalent than externalizing problems, such as aggression and delinquent behaviour [1].

Within our systematic review we found suggestion that children had higher rates of body concern, depression, anxiety, somatization, difficulty in relating to others and greater emotional and behavioural problems [5,7,12,15,19,20,23,25]. Children also perceived their families as being less cohesive with greater tension and isolation, as compared with the general population [14,22]. Furthermore, uncertainty regarding the future, as well as illness exacerbation, posed a degree of fear and anxiety in children [25,40]. Caregiving roles and the stigma attached to a parent’s MS were also sources of stress for children [20]. Specifically for adolescents, greater family responsibilities were linked to fewer social relationships and higher distress [19,20,24]. However, several studies found no measurable effect (negative or positive) of having a parent with MS [5,8,16,18], and a few found some positive effects, such as higher personal competence [13,14].

This pattern of positive and negative outcomes could reflect the costs and benefits associated with caregiving that is also evident in adult caregivers [41]. Some children in our review described pride in their caregiving abilities, as these children completed tasks above those of their peers [10]. However, there are circumstances, when children may feel they have no choice but to become caregivers, and that can intensify the overall family stress [19,38]. Children are more prone to becoming caregivers in single-parent families, low-income families, families who do not have access to home care support, and families with little social support [5,42].

Our systematic review highlighted that one of the factors associated with poor adjustment in children was their limited knowledge and understanding of MS [17,21]. This concurs with other studies which indicate that it is important to provide children information about MS, tailored to their developmental level, as this lack of information has caused some children to believe that their own or other people’s behaviour affected their parents’ illness [43,44]. Young children, in particular, appear to have a need for information that is currently not being met [43]. Children may need better information on the etiology of MS and be reassured that the risk for them to contract MS is minimal [43]. Children who are unaware of their parent’s illness may display high levels of anxiety and distress as they witness family tension without being aware of its source [21]. Interestingly, educated mothers are less likely to provide information regarding their illness to their children [21]. This observation is worthy of further investigation, especially as several studies show that MS patients who participate in research have a higher socioeconomic status compared with other chronic illness patients, which could potentially lead to selection and reporting bias [5,45].

In our systematic review, studies reported that family dysfunction and lack of social support were associated with a child’s externalizing problems, while the severity of impairment of the ill mother predicted children’s internalizing problems [7,14]. This is consistent with other studies which showed that lower level of depression in the affected parent has been related to positive coping ability of the children [46]. Likewise, the coping ability of the healthy parents appeared to be a strong predictor of whether children successfully cope with the disease [46,47]. Nonetheless, no study in our systematic review looked at fatigue as a risk factor, whilst fatigue has been shown to be one of the most common, yet “hidden”, symptoms of MS [48]. Parents with MS identified fatigue as one of the primary problem that interferes with important parenting functioning, ranging from difficulties in being involved with day-to-day activities, to lack of patience [20]. Furthermore, in a group of patients with different chronic illnesses, including MS, one study demonstrated that maternal fatigue potentially mediates some of the relationship between maternal depression and maladaptive child outcomes [45]. Other factors which emerged as potentially influencing a child’s adjustment to parental illness from the included studies were: gender of the parent and the child [20,46,47], children’s age and developmental stage [10,19], level of social support [10], physical condition or disability caused by the disease [19], single parenthood and family environment [10,15,19,23].

Interpretation of our systematic review is constrained by limitations in the original studies, particularly exposure assessment and potential sources of bias. First, none of the studies included in the present systematic review used objective measures of child psychosocial adjustment. Second, studies failed to account for important confounding or to provide baseline characteristics of the participants that limit the generalizability of the findings. Third, many studies did not include a comparison group, which is a critical piece in shedding light on whether the patterns of findings in the studies are specific to children who have a parent with an illness. Last, relying on cross-sectional design fails to disentangle the interactions between normal child developmental variations and the variations produced by the progressive nature of MS, such as difficulties in the transition to adulthood. This is particularly relevant given that disability in MS can often be minimal in the early stages of the disease, and the overall lifespan may not be affected as with other chronic diseases. Furthermore, to explore if there is an MS specific characteristics that influence children’s development, it would be of interest to investigate the psychosocial wellbeing of children with MS parents as compared to other chronic diseases.

To overcome many of the deficiencies raised above, we recommend a population-based approach with inclusion of a representative comparison group to avoid selection biases that may have limited many studies. In addition, rigorous, objective, and well-validated measurement tools are needed, of which several are available, namely, the Early Child Development Instrument [49], or the Child Behaviour Checklist [27]. Along with this, measurement of appropriate confounders or explanatory variables, such as socioeconomic status, gender of the child or the marital status of the parents should be considered. Ideally these would be combined with clinical characteristics of the affected parents, such as disease duration, level of disability or presence of comorbidity, to populate a large study-specific dataset of patients and their children. Findings of these studies would help to inform policy making for healthier communities and assist us in developing and evaluating family centered interventions to improve child and family outcomes [50].

### Potential limitations of the review process

Some of the following potential limitations are common across systematic reviews, such as publication bias and study selection process. We sought to mitigate these by having two independent reviewers, and by checking reference lists of previously published reviews and articles retrieved in the search for studies that we might have missed. Despite these measures, the selection and qualitative synthesis of eligible studies can still be a rather subjective process. However, by using a standardized form to extract the data, and assessing methodological quality using a validated checklist, we strived to maximize the objectivity in our search strategy. For observational and epidemiological study designs, there is currently a lack of consensus regarding the most appropriate methodology for assessing quality in the context of a systematic review [51]. Although the GATE tool is an excellent tool to critically appraise different types of studies, it does not assign a grade or score to studies and therefore its use and validity might be limited [52,53].

## Conclusion

Exploring the relationship between parental MS and a child’s psychosocial adjustment is challenging. Due to the relatively few studies of high methodological quality, it is difficult to draw strong evidenced-based conclusions from the present literature and thus more extensive and higher quality research in this area is greatly needed. From the limited available evidence, it appears that exposure to parental MS may put children at a higher risk of psychosocial problems compared with children with parents not living with a chronic disease. Although the few studies examining the impact of parental chronic disease on children’s development and health represent important first steps, many have serious methodological limitations, particularly with respect to ascertaining individuals with definite MS, and potential sources of bias, such as failure to adjust for important demographic variables, i.e. socio-economic status, lack of a suitable comparison group and sub-optimal data collection. To gain further insight and to assess this relationship accurately we need more population-based studies using objective measures of developmental health, using reliable and valid measurement instruments. Further research is needed before appropriate evidenced-based recommendations can be made, however, it appears pragmatic to advise healthcare professionals, and community partners, such as educators, patient group and policy makers, to be cognizant of the broad impact of chronic parental illness on the developing child.

## Abbreviation

MS: Multiple Sclerosis; GATE: Graphic Appraisal Tool for Epidemiology; EDSS: Kurtzke’s Expanded Disability Status Scale.

## Competing interests

The authors’ declare that they have no competing interests.

## Authors’ contributions

NR was responsible for the design and conceptualization of the study, analysis and interpretation of the data, drafting and revising the manuscript. RN participated in analysis and interpretation of the data. RAM assisted in interpretation of the data and revising the manuscript. WTM assisted with interpretation of the data and revising the manuscript. HT participated in analysis and interpretation of the data and helped to draft the manuscript. All authors read and approved the final manuscript.

## Pre-publication history

The pre-publication history for this paper can be accessed here:

http://www.biomedcentral.com/1471-2377/14/107/prepub

## Supplementary Material

Additional file 1 Search strategies and results. Click here for file

Additional file 2 Data extract form. Click here for file
