# Supplementary material for: Children and adolescents adjustment to parental multiple sclerosis: a systematic review
Source: BMC Neurol. 2014 May 19;14:107. doi: 10.1186/1471-2377-14-107 (PMC4040480; doi:10.1186/1471-2377-14-107)
Supplement: Additional file 1 — Search strategies and results. [file 1471-2377-14-107-S1.doc]

**APPENDIX 1 - Search Strategies and Results**

Database: Ovid MEDLINE(R) In-Process & Other Non-Indexed Citations and Ovid MEDLINE(R) <1946 to Present>

Search Strategy:

--------------------------------------------------------------------------------

1 multiple sclerosis.mp. or exp Multiple Sclerosis/ (51343)

2 disseminated sclerosis.mp. or exp Multiple Sclerosis/ (40988)

3 sclerosis, disseminated.mp. or exp Multiple Sclerosis/ (40884)

4 sclerosis, multiple.mp. or exp Multiple Sclerosis/ (40934)

5 multiple sclerosis, acute fulminating.mp. or exp Multiple Sclerosis/ (40882)

6 exp Multiple Sclerosis, Relapsing-Remitting/ or Demyelination, demyelinization.mp. (2978)

7 exp Multiple Sclerosis/ or Dorsal sclerosis.mp. (40883)

8 exp family/ (220549)

9 exp Parents/ (65820)

10 exp Parent-Child Relations/ (43212)

11 exp "Child of Impaired Parents"/ (3814)

12 exp Nuclear Family/ (80078)

13 exp Caregivers/ (18985)

14 8 or 9 or 10 or 11 or 12 or 13 (234808)

15 1 or 2 or 3 or 4 or 5 or 6 or 7 (51460)

16 14 and 15 (540)

***************************
