# Supplementary material for: Children and adolescents adjustment to parental multiple sclerosis: a systematic review
Source: BMC Neurol. 2014 May 19;14:107. doi: 10.1186/1471-2377-14-107 (PMC4040480; doi:10.1186/1471-2377-14-107)
Supplement: Additional file 2 — Data extract form. [file 1471-2377-14-107-S2.doc]

**APPENDIX 2 – Data Extract Form**

# Review Title:

Reviewer: _______________________ Date Reviewed: _________

Study ID: __________

| Study Information | Author & Year: | | Citation: | | |  |
| --- | --- | --- | --- | --- | --- | --- |
| Type of Study |  RCT  Controlled Clinical Trial  Quasi RCT Retrospective Cohort  Case-Control  Case Series  Other | | | | |  |
|  | Inclusion | | | Exclusion | |  |
| - Eligible Participants: - Exposure: - Comparisons: - Outcomes: |  | | |  | |  |
| **Study Element** | | **Description** | | | **Risk of Bias (Low, High, Unknown)** | |
| **Participants:**   - Age of Children:  Study Setting: - Geographical region: - Were the subjects asked to participate in the study representative of the entire population from which they were recruited? - Were study subjects in different exposure groups recruited from the same population? | |  | | |  | |
| **Exposure status:**   - Were exposure and comparison measures well described and valid? Were exposure and comparison factors measured prior to outcomes - Did exposure/comparison status change during follow-up? - Were case definitions well described and valid? - Were other exposures similar in both groups during follow-up? Were all participants accounted for at study conclusion? | |  | | |  | |
| **Comparisons:**   - Selected cases/ comparators representative of all eligible cases/comparators? - Eligible population well described? Were relevant personal characteristics in participants reported? | |  | | |  | |
| **Outcomes:**   - How were outcomes measured? - Blinded outcome measurement? - Were selected outcomes measurable and meaningful? - Was follow-up time meaningful? | |  | | |  | |

| **Analysis:**   - What type of analysis was performed? - Was the analysis appropriate? - Were covariates/ - Confounders measured and adjusted for? Effect estimates given or calculable? - Precision of effect estimates given or calculated? |  |  |
| --- | --- | --- |
| **Results:**   - Study internally valid? - Are results of sufficient magnitude & precision? - Can the generalizability of the results be determined? |  |  |
